# Pharmacogenomic insights in psychiatric care: uncovering novel actionability, allele-specific *CYP2D6* copy number variation, and phenoconversion in 15,000 patients

**DOI:** 10.1038/s41380-024-02588-4

**Published:** 2024-05-23

**Authors:** Jai N. Patel, Sarah A. Morris, Raul Torres, Brooke Rhead, Chris Vlangos, Daniel J. Mueller, Lisa C. Brown, Hailey Lefkofsky, Muneer Ali, Francisco M. De La Vega, Kathleen C. Barnes, Anthony Zoghbi, Joseph D. Stanton, Marcus A. Badgeley

**Affiliations:** 1 Department of Cancer Pharmacology & Pharmacogenomics, Levine Cancer Institute, Atrium Health, Charlotte, NC USA; 2 Tempus AI, Inc., Chicago, IL USA; 3 Pharmacogenetics Research Clinic, Campbell Family Mental Health Research Institute, Centre for Addiction and Mental Health, Toronto, ON Canada; 4 Department of Psychiatry, University of Toronto, Toronto, ON Canada; 5 Great Scott! Consulting, New York, NY USA; 6 Department of Psychiatry and Behavioral Sciences, Baylor College of Medicine, Houston, TX USA

**Keywords:** Biomarkers, Genetics

## Abstract

Pharmacogenomic testing has emerged as an aid in clinical decision making for psychiatric providers, but more data is needed regarding its utility in clinical practice and potential impact on patient care. In this cross-sectional study, we determined the real-world prevalence of pharmacogenomic actionability in patients receiving psychiatric care. Potential actionability was based on the prevalence of CYP2C19 and CYP2D6 phenotypes, including CYP2D6 allele-specific copy number variations (CNVs). Combined actionability additionally incorporated CYP2D6 phenoconversion and the novel *CYP2C*-TG haplotype in patients with available medication data. Across 15,000 patients receiving clinical pharmacogenomic testing, 65% had potentially actionable CYP2D6 and CYP2C19 phenotypes, and phenotype assignment was impacted by *CYP2D6* allele-specific CNVs in 2% of all patients. Of 4114 patients with medication data, 42% had CYP2D6 phenoconversion from drug interactions and 20% carried a novel *CYP2C* haplotype potentially altering actionability. A total of 87% had some form of potential actionability from genetic findings and/or phenoconversion. Genetic variation detected via next-generation sequencing led to phenotype reassignment in 22% of individuals overall (2% in CYP2D6 and 20% in CYP2C19). Ultimately, pharmacogenomic testing using next-generation sequencing identified potential actionability in most patients receiving psychiatric care. Early pharmacogenomic testing may provide actionable insights to aid clinicians in drug prescribing to optimize psychiatric care.

## Introduction

Nearly half of patients diagnosed with major depressive disorder (MDD) do not achieve adequate symptom reduction with traditional approaches to antidepressant selection [1, 2]. Treatment selection is partly trial-and-error, and unsuccessful medication trials prolong disabilities associated with depression [3]. Medication selection considerations include symptom characteristics, drug interactions, adverse event profiles, response to prior treatment(s), and other comorbidities. Genetic variation between patients has also been shown to affect responses to antidepressants [4–6], which can be tested with a pharmacogenetic (PGx) assay. Accordingly, several guidelines exist for evidence-based prescribing recommendations from the Food and Drug Administration (FDA) and the Clinical Pharmacogenetics Implementation Consortium (CPIC) [7–12]. One study showed that a PGx assay identified ≥1 gene-drug interactions (GDI) for a considered medication in more than half of patients [13]. Notably, gene-drug-drug interactions (GDDI), known as phenoconversion, can also exist where medications can inhibit or induce enzymes involved in drug metabolism and alter their baseline metabolizer phenotype [14]. Despite the extensive evidence-based recommendations for GDIs and GDDIs, some commercial assays report phenotypes that are not consistent with CPIC guidelines [15–17].

The guidelines for PGx testing and phenoconversion are supported by rigorous evidence and processes; however, updates to these guidelines do not keep pace with the emergence of new evidence [7, 8, 14, 18–20]. New variants can be discovered with next-generation sequencing (NGS) but need to be validated in other studies using comparable technology and methods [21]. For instance, one haplotype recently described involves two loci between *CYP2C18* and *CYP2C19* that affects selective serotonin reuptake inhibitor (SSRI) metabolism - called the “*CYP2C* haplotype” [22]. Patients may be classified as having normal *CYP2C19* metabolism according to existing assays/guidelines, but carrying the *CYP2C* haplotype alleles rs2860840 C>T and rs11188059 G>A (“*CYP2C*-TG”) can increase their metabolism from normal to rapid or ultrarapid [22], changing potential actionability, although this was not reproduced in a subsequent study [23].

Copy number variations (CNVs) can lead to multiple alleles due to the variable number of copies, and when the alleles have different activity scores, the overall metabolism depends on which allele is copied. This is common for *CYP2D6*, and there are guidelines for assigning metabolic phenotype based on allele activity scores and assigned CNVs [24].

Most clinical PGx assays and commercial assays (targeted single-nucleotide polymorphism [SNP] assays) likely only test haplotypes described in guidelines [25–27] and cannot accurately assign *CYP2D6* CNVs to an allele [21]. NGS provides the opportunity to interrogate *CYP2D6* allele-specific CNVs and novel variations like the *CYP2C* haplotype.

Here, we evaluated the prevalence of guideline-based PGx actionability in a large, real-world cohort of patients receiving psychiatric care who were referred for clinical PGx testing using NGS. Secondarily, we determined the frequency of *CYP2D6* CNVs in which identification of allele-specific CNVs altered phenotype assignment, the frequency of phenoconversion, and potential impact of the novel haplotype (*CYP2C*-TG) on CYP2C19 phenotype assignment. Based on these findings, we estimated the combined potential actionability of PGx testing in patients receiving psychiatric care.

## Methods

### Study design and data collection

A subset of de-identified records from patients who underwent Tempus nP PGx testing (Tempus AI, Inc, Chicago, IL) between April 2020 and November 2022 as part of their psychiatric care was selected for retrospective analysis. Patient demographics, diagnoses, and medications were obtained from order forms or attached progress notes. This study was deemed exempt from institutional review by the Advarra Institutional Review Board (Pro00072742).

### Metabolizer phenotypes and CNV allele assignment

Patients’ saliva specimens were assayed with the Tempus nP laboratory-developed test, which combines NGS whole-exome sequencing (WES) and Agena MassARRAY^®^ in a College of American Pathologists/Clinical Laboratory Improvement Amendments-accredited laboratory (Tempus AI, Inc., Chicago, IL, USA) [19]. Detailed methodology describing sequencing equipment, bioinformatic processing, and validation are provided in the supplementary methods. Star alleles and metabolizer phenotypes were based on clinical guidelines from CPIC, the Dutch Pharmacogenomics Working Group (DPWG), and the Association for Molecular Pathology (AMP). Genetic results were reviewed and finalized by a board-certified clinical geneticist.

Genomic DNA was used for analysis of *CYP2D6* single-nucleotide variations (SNVs) and small insertions/deletions (indels) by NGS. Data synthesized from NGS and MassARRAY^®^ were used in combination to assign *CYP2D6* CNVs to a specific allele. The impact of *CYP2D6* CNV allele assignment on metabolism phenotype was assessed based on activity scores as described in CPIC and DPWG consensus recommendations [24] and is referred to as “allele-specific CNV-actionability” when allele copy number assignment disambiguates patients among metabolism phenotypes with different therapy guidance (see section “PGx Actionability” below). Tempus’ system for personalized psychiatric disorder treatment is described in U.S. patent 10978196 [28].

### PGx actionability: potential and clinical

CYP2D6 and CYP2C19 phenotypes were defined as “actionable” based on CPIC guidelines [7, 8, 11] (regardless of classification of recommendation) and the FDA Table of Pharmacogenetic Associations [12] for which the Data Support Therapeutic Management Recommendations (Section 1) (hereafter referred to as the “FDA Table”; Supplementary Table S1). Two levels of actionability were defined:

- “Potentially actionable”: presence of CYP2D6 or CYP2C19 phenotypes associated with GDIs in the FDA Table or CPIC guidelines (regardless of medication(s) prescribed or considered), including potentially actionable CYP2D6 or CYP2C19 phenotypes.
- “Clinically actionable”: presence of a GDI (Supplementary Table S1) based on prescribed or considered medications in patients with medication data available at time of testing.

### Genetic ancestry and race/ethnicity imputation

Continental genetic ancestry proportions were predicted from WES data using a supervised version of the ADMIXTURE algorithm [29]. After conditioning variants on having adequate sequencing coverage on the Tempus xE and nP assays, data from the 1000 Genomes Project [30] and the Simons Genome Diversity Project [31] were used to identify a set of 6711 ancestry-informative markers (AIMs) varying in frequency among individuals from five continental regions: Africa (AFR), the Americas (AMR), Europe (EUR), East Asia (EAS), and South Asia (SAS). For each exome sequence input, the model yields estimates of the proportions of ancestry arising from each of the five continental regions.

Many patients have a mixture of continental genetic ancestries representing the genetic similarities shared between different groups. In contrast, race and ethnicity are not biological, but rather social/cultural categorizations that patients are assigned to and/or identify with [32]. We imputed race/ethnicity groups from genetic admixtures using proportions observed in patients with solid-tumor sequencing and stated race/ethnicity labels at Tempus AI [33], which are consistent with those reported for African American/Black and Hispanic/Latino groups in the United States [34]. Race/ethnicity imputation cutoffs were selected heuristically by creating mutually exclusive groups. Imputed race/ethnicity cutoffs are as follows: >20% AFR, <10% AMR, and >70% combined AFR and EUR ancestry were classified as Non-Hispanic Black (hereafter referred to as “Black”), >10% AMR and >70% combined AMR, EUR, and AFR ancestry were classified as Hispanic/Latino, >70% combined EAS and SAS ancestry were classified as Asian, >80% EUR and <10% AMR ancestry were classified as Non-Hispanic White (hereafter called “White”), and all others were classified as Complex Admixture (hereafter called “Admixed”) [35].

### Phenoconversion

Phenoconversion indicates that the patient’s metabolism phenotype after accounting for interacting medications is different from that assigned by genotype alone [36]. The Indiana University Flockhart Table [37] (Supplementary Table S2) was utilized to estimate *CYP2D6* phenoconversion in patients with medication data where medications were categorized as strong or moderate inhibitors [36]. CYP2D6 activity score was defined as 0 or multiplied by 0.5 for strong or moderate inhibitors, respectively.

### Genotype imputation of a novel CYP2C haplotype

Only 1 of 2 loci that comprise the *CYP2C*-TG haplotype is targeted by the nP NGS-enhanced assay, therefore patients’ genotypes were imputed and phased at these loci using off-target sequencing reads and a reference panel of haplotypes. The imputation algorithm, GLIMPSE [38], is described in the supplementary methods. Haplotype imputation methods and software commands are provided in the “Haplotype Imputation Commands” section. Imputation performance was evaluated using the HG001 reference sample from the National Institute of Standards and Technology (NIST) (described in the supplementary methods and results).

### Statistical analysis

Confidence intervals for frequency estimates were computed using bootstrap analysis. For each patient group, we randomly sampled with replacement the number of patients originally in the group 1000 times, computed the 1000-sample frequencies of a phenotype or actionability, and then reported (or plotted) 2.5th and 97.5th percentiles.

To compare frequencies of actionability among race/ethnicities, we performed chi-squared 5-sample, 2-sided proportion tests. If found to be significant (*P* < 0.05), then pairwise 2-sided proportion-tests between each pair of imputed race/ethnicities were applied and corrected for multiple hypotheses with Holm using the R stats package [39].

### Combined actionability of PGx results, phenoconversion, and novel haplotype

We prioritized and combined actionability sources according to assay features for patients with medication and NGS data to determine initial actionability of each patient. Initial actionability was summed to determine the combined actionability of the cohort, where “initial actionability” was defined in the following order:

1. Did the patient have *CYP2C19* or *CYP2D6* potential actionability (accounting for allele-specific CNV-actionability)?
  - NGS was required to identify the initial actionability when:
    i. *CYP2D6 was* actionable with allele-specific CNV-actionability, and *CYP2C19* was not actionable; or
    ii. There was no PGx actionability and ruling out *CYP2D6* actionability required allele-specific CNV (e.g., a *1/*4 patient whose CNV was assigned to *1 resulting in a normal metabolizer phenotype).
2. Did the remaining patients carry *CYP2C*-TG?
  - NGS was required to identify the CYP2C-TG haplotype and identify patients that were initially CYP2C19 normal metabolizers but found to carry the *CYP2C*-TG actionable haplotype.
3. Did the remaining patients have *CYP2D6* phenoconversion from non-actionable to actionable?

## Results

### Patient characteristics

**Fig. 1:**
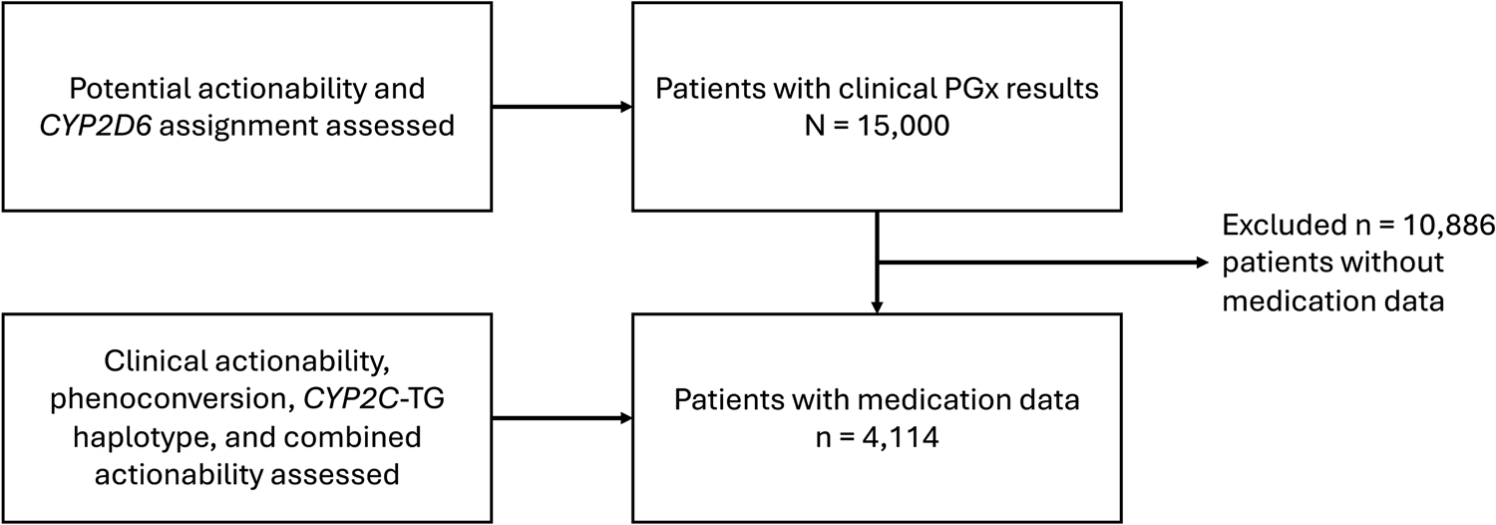
Consort diagram. Patients with clinical PGx results were assessed for potential actionability of results and *CYP2D6* allele-specific copy number variation actionability. Patients with clinical PGx results and medication data were assesed for clinical actionability of results, *CYP2D6* phenoconversion, presenence of a novel *CYP2C-TG* haplotype. The presence of clinical actionability, *CYP2C-TG* haplotype, or phenoconversion was assessed to determine the combined actionability of the cohort with PGx results and medication data.

Across 15,000 PGx-tested patients (median age=30 years, interquartile range 18–44 years, 63% female), the most common diagnoses were MDD (44%), generalized anxiety disorder (38%), and attention-deficit hyperactivity disorder (19%) (Table 1). Fifty-two percent of patients previously tried ≥1 prior medication. Analyses on clinical PGx testing were done in the overall cohort of 15,000 patients, whereas Research Use-Only analyses of phenoconversion and CYP2C haplotypes were conducted in the 4,114-patient subset with available medication and NGS data (Fig. 1).

**Table 1:** Patient demographics, diagnoses, and CYP2D6/CYP2C19 phenotypes.

Characteristics	
Age (median, range)	30 (0.5–98)
Gender	N (%)
Male	5296 (35%)
Female	9493 (63%)
Not Stated	211 (2%)
Imputed race/ethnicity	
White	12,250 (82%)
Hispanic/Latino	1077 (7%)
Black	964 (6%)
Complex	371 (2%)
Asian	338 (2%)
Diagnosis	
MDD	6659 (44%)
GAD	5708 (38%)
ADHD	2878 (19%)
BPD	1231 (10%)
PTSD	1103 (8%)
SCZ	79 (<1%)
CYP2C19 Metabolizer Phenotypes	
Poor	420 (3%)
Intermediate	3998 (27%)
Normal	6041 (40%)
Rapid	3573 (24%)
Ultrarapid	995 (7%)
CYP2D6 Metabolizer Phenotypes	
Poor	873 (6%)
Intermediate	5849 (39%)
Normal	7770 (52%)
Ultrarapid	508 (3%)

*MDD* major depressive disorder, *GAD* Generalized anxiety disorder, *ADHD* Attention deficit hyperactivity disorder, *BPD* bipolar disorder, *PTSD* Post-traumatic stress disorder, *SCZ* Schizophrenia.

### Potential actionability from CYP2C19 and CYP2D6 phenotypes

Sixty-five percent of patients had a potentially actionable CYP2C19 and/or CYP2D6 phenotype associated with decreased efficacy or increased risk of toxicity for ≥1 psychiatric medication according to CPIC and/or the FDA Table (Supplementary Table S1).

### Clinical actionability based on the ordering clinician’s medications

**Fig. 2:**
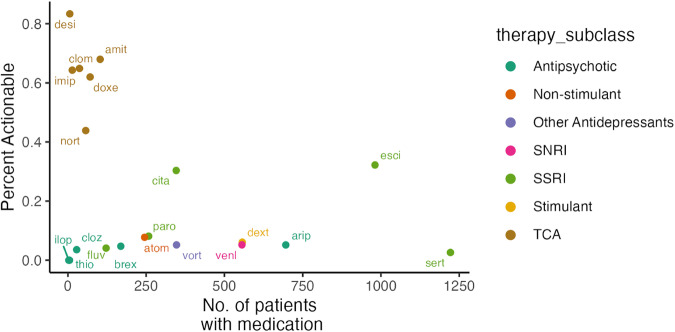
Clinical actionability for medications attempted and considered (combined) at the time of PGx testing. Each point represents a psychiatric medication with PGx prescribing guidance. The *x*-axis represents how many patients had the medication, and the *y*-axis shows how frequently those patients had clinically actionable PGx results. amit amitriptyline, arip aripiprazole, atom atomoxetine, brex brexpiprazole, cita citalopram, clom clomipramine, cloz clozapine, desi desipramine, deut deutetrabenazine, dext dextroamphetamine, doxe doxepin, esci escitalopram, fluv fluvoxamine, ilop iloperidone, imip imipramine, nort nortriptyline, paro paroxetine, sert sertraline, valb valbenazine, venl venlafaxine, vort vortioxetine.

Across 15,000 patients, 2215 had prior or current psychiatric medication information available at the time of PGx testing. Of these, 1663 patients (74%) attempted medications metabolized by CYP2D6 and/or CYP2C19 enzymes, where 360 (22%) had ≥1 clinically actionable phenotype. Medication data was also available on 2478 patients for whom the clinician was considering a new psychiatric medication. Of 1674 patients considering medications metabolized by CYP2D6 and/or CYP2C19 enzymes (68%), 295 (18%) had ≥1 clinically actionable phenotype. SSRIs were the most common attempted (n = 1,594) or considered (n = 1,334) medication class (Supplementary Table S3, Supplementary Table S4), with citalopram and escitalopram having the most frequent GDIs (32% and 30% of patients reporting medications, respectively), and sertraline being the least frequent with FDA/CPIC guidance (3%) (Fig. 2). Although tricyclic antidepressants (TCAs) were not commonly attempted (n = 132) or considered (n = 123), they were most likely to interact (59%) with the CYP2D6 and/or CYP2C19 phenotypes (Supplementary Table S5, Supplementary Table S6). Aripiprazole, dextroamphetamine/amphetamine, and venlafaxine had similar actionability rates (5%, 6%, and 5% of patients, respectively).

### Imputed race and ethnicity

**Fig. 3:**
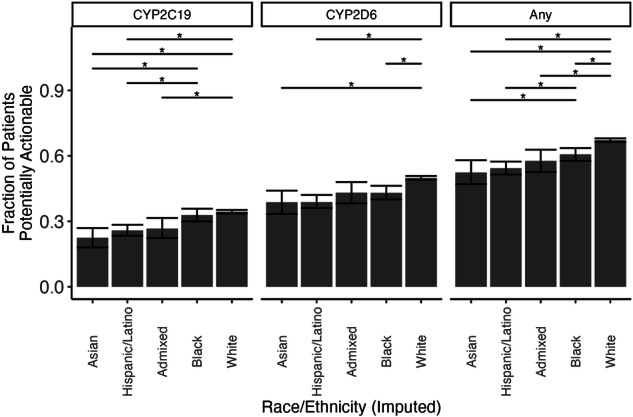
Potential actionability by imputed race/ethnicity and gene in 15,000 patients. The 95% confidence limits for each race/ethnicity and gene’s potential actionability are indicated with error bars and were estimated with nonparametric bootstrapping. Proportion tests between pairs of race/ethnicity are displayed as horizontal lines with stars “*” above pairs that are significantly different (*P* < 0.05) and sorted from most significant to least significant top to bottom.

Among the cohort, 82% were imputed as White, 7% Hispanic/Latino, 6% Black, 2% Asian, and 2% complex admixed (Supplementary Fig. 1). Potential actionability rates were highest for Whites (*CYP2D6* = 50%, *CYP2C19* = 34%) and Blacks (*CYP2D6* = 43%, *CYP2C19* = 33%), and lowest for Asians (*CYP2D6* = 39%, *CYP2C19* = 22%) (Fig. 3). *CYP2C19* potential actionability was significantly higher for Whites than Hispanic/Latinos, Asians, and complex admixed (*P* ≤ 0.02); *CYP2D6* potential actionability was significantly higher for Whites than Hispanic/Latinos, Blacks, and Asians (*P* ≤ 6e-4). Overall, Whites had significantly higher actionability rates than all other groups (*P* ≤ 9e-4) and Blacks had higher actionability rates than Hispanic/Latino and Asian groups (*P* = 0.05).

### CYP2D6 copy number variations

Of 15,000 patients tested, 1,104 (7%) harbored *CYP2D6* CNVs, and 338 (30%, or 2% of the total population) had a genotype where knowledge of allele-specific *CYP2D6* CNVs would change the phenotype and corresponding actionability. The most common *CYP2D6* genotypes requiring allele-specific CNVs to assign correct phenotype were *1/*4, *2/*4, *2/*41, *4/*35, *35/*41, *10/*41, *1/*41, and *1/*6 (Table 2). The rate of *CYP2D6* CNVs and allele-specific CNV-actionability were significantly different among imputed races (5-sample chi-square *P* = 2e-16 and 2e-5, respectively). *CYP2D6* CNV rate was highest in Asians and lowest in Whites, while the rate of allele-specific CNV actionability was highest in Blacks (4%) (Supplementary Table S7).

**Table 2:** Most common *CYP2D6* diplotypes with copy number variation (CNV).

CNV diplotype	No. of patients	Phenotype	Phenotype depends on CNV assignment	Alternative phenotype
*1/*2xN	146	Ultrarapid	No	
*1/*1xN	138	Ultrarapid	No	
*1/*4xN	112	Intermediate	Yes	Normal
*1/*10xN	58	Normal	No	
*4/*4xN	56	Poor	No	
*2/*2xN	56	Ultrarapid	No	
*2xN/*4	52	Normal	Yes	Intermediate
*2/*4xN	42	Intermediate	Yes	Normal
*10/*10xN	41	Intermediate	No	
*1xN/*4	36	Normal	Yes	Intermediate
*2xN/*41	30	Ultrarapid	Yes	Normal
*1xN/*2	30	Ultrarapid	No	

This table shows the most common diplotypes that were observed in those patients found to have CNVs (of the 15,000-patient PGx cohort). Accurate CNV assignment was performed using NGS + MassARRAY^®^. The Phenotype field shows the codified phenotype with allele assignment, and the Alternative Phenotype shows what the phenotype would be if the other allele had been copied (i.e., when the phenotype depends on accurate CNV assignment).

### Phenoconversion

Among 4114 patients with any reported medications, 1980 (48%) were reported to receive medications that were moderate or strong CYP2D6 inhibitors, most commonly bupropion (n = 1002 patients) and fluoxetine (n = 952). Of those receiving moderate or strong CYP2D6 inhibitors, the CYP2D6 metabolism phenotype and potential actionability changed in 1734 patients (87%) – or 42% of all patients with reported medications (relative to the genotype-based phenotype) (Supplementary Table S8).

### CYP2C haplotypes

Genotype imputation at individual *CYP2C* loci in the 4,114 patients with medication data predicted alternative frequencies matching the National Institutes of Health (NIH) dbSNP database: 33% rs2860840 C>T [40] and 13% rs11188059 G>A [41]. On average, there were NGS results at 1,436 (standard deviation (SD) = 196) of the 7220 linked SNPs in the region (region = chr10:96375267-98547144; mean effective coverage = 0.2×; SD = 0.03x).

The overall frequency of the *CYP2C*-TG haplotype was 36%. Of patients with a *CYP2C19* *1/*1 diplotype (40%), 51% were expected to have at least one *CYP2C*-TG allele. Therefore, should the CYP2C haplotype be validated and considered actionable in future guidelines, then approximately 21% of all patients could experience a change in CYP2C19 actionability from normal metabolizer to rapid or ultrarapid metabolizer when this variant is interrogated. Additionally, *CYP2C*-TG frequencies differed significantly among imputed race/ethnicities. Black patients had the lowest frequency (14%) compared to Asian (34%), White (37%), and Hispanic/Latino (46%) (Supplementary Table S9, Supplementary Table S10). Hispanics had a higher frequency of altered phenotypes (30%) than Whites (21%) and Blacks (9%) (Supplementary Table S11).

### Combined actionability in patients with medication data

Considering the 4,114 patients with medication and WES data available, 66% had a potentially actionable CYP2D6 or CYP2C19 phenotype with 2% requiring NGS to confirm allele-specific *CYP2D6* CNVs (Table 3). Of the remaining 34% of patients, an additional 10% did not have a potentially actionable CYP2D6 or CYP2C19 phenotype but were expected to carry the *CYP2C*-TG allele (based on WES), increasing the total potential actionability to 76%. Further, an additional 11% of patients had a drug interaction resulting in CYP2D6 phenoconversion, culminating in a final combined actionability of 87%. Overall, 11% of patients had initial actionability, and 22% had some actionability that was detected with the NGS-enhanced PGx platform.

**Table 3:** Initial actionability attributable to established PGx guidelines, the emerging *CYP2C* haplotype, and phenoconversion.

Initial actionability	No. of patients	% of Patients	% of Actionability that is NGS dependent
PGx^a^	2732	66	2
CYP2C^b^	402	10	100
Phenoconversion^c^	464	11	1
None^d^	516	13	1
Any^e^	3598	87	11

^a^“PGx” indicates the patient had a potentially actionable CYP2D6 or CYP2C19 phenotype.

^b^“CYP2C” indicates the patient was expected to carry the CYP2C-TG allele.

^c^“Phenoconverison” indicates the patient had a reported medication resulting in CYP2D6 phenoconversion.

^d^“none” indicates the patient had no potentially actionable phenotype, did not harbor the CYP2C haplotype, and did not have phenoconversion.

^e^“any” indicates the patient had at least one source of actionability among PGx, novel CYP2C, and/or phenoconversion.

## Discussion

To our knowledge, this is the first study to evaluate PGx actionability of *CYP2D6* and *CYP2C19* variants in a large real-world cohort of patients receiving NGS-based clinical PGx testing in the US. In this study of 15,000 patients, the majority (65%) had a potentially actionable CYP2D6 or CYP2C19 phenotype which corresponded to a GDI based on CPIC guidelines and the FDA Table Section 1. Roughly half of the patients who had medication data available reported taking a strong or moderate CYP2D6-inhibiting medication resulting in phenoconversion and altered metabolism phenotype from baseline. *CYP2D6* CNVs were observed in 7% of patients and the NGS-based PGx test allowed for allele-specific CNV assignment, leading to a change in CYP2D6 phenotype assignment in almost one-third of these patients. Using NGS to also impute a novel *CYP2C* haplotype demonstrated that half of the patients currently assigned a CYP2C19 normal metabolizer phenotype carry this novel haplotype and may be predicted to metabolize certain antidepressant medications faster than normal, if validated in subsequent studies.

The prevalence of actionable phenotypes observed in our predominantly European ancestry cohort is similar to previous studies of individuals of European ancestry [42], and marked differences in *CYP2D6* CNV rates (~2-fold) have previously been observed between geographic cohorts [43, 44]. The predominance of White patients in this cohort may be exacerbated by racial disparities in the U.S. in receiving PGx testing to guide their care [45, 46]. Actionable phenotypes were observed across all race and ethnicity categories in this cohort, albeit at lower frequencies in minority groups. Notably, most alleles associated with drug response have been discovered primarily in patients of European ancestries [47], which may inform the alleles included on various panels, thus limiting their actionability to cohorts of other ancestries [48].

In the subset of patients with medication data, 22% and 18% of patients had a GDI with their attempted and considered medications, respectively. SSRIs, specifically citalopram and escitalopram, were the most common medications affected by PGx, as these are commonly prescribed medications with prescribing guidance for all non-normal *CYP2C19* metabolizers [7, 12, 20]. The most prescribed SSRI, sertraline, had 10x lower rates of actionability than (es)citalopram because there is only guidance for *CYP2C19* poor metabolizers. Although TCAs are not frequently prescribed, this class of medications is most likely to be clinically actionable since many TCAs are impacted by both CYP2C19 and CYP2D6 metabolism [8]. Due to marked differences in actionability rates between medications, healthcare providers and systems may consider PGx testing more strongly when patients are eligible for medications with high actionability rates.

We were able to assign CNVs to a specific *CYP2D6* allele to improve accuracy in assigning genotype and phenotype. In this study, nearly one-third of patients with CNVs would have been assigned a phenotype range, assigned a potentially inaccurate phenotype, or marked as indeterminant in the absence of allele-specific CNV assignment. Precise genotypic and phenotypic assignment are crucial to identify patients with actionable results and optimize PGx-guided medication prescribing for the more than 20 psychiatric-related medications metabolized by CYP2D6.

Based on the CPIC guideline for SSRIs published in 2015, sertraline should be initiated at standard dose except for CYP2C19 poor metabolizers; however, there is emerging evidence that additional haplotypes and genes can impact the metabolism of sertraline and other SSRIs. The *CYP2C*-TG haplotype was reported in patients previously identified as CYP2C19 normal metabolizers to be associated with decreased sertraline and escitalopram levels similar to CYP2C19 rapid/ultrarapid metabolizers, therefore potentially requiring higher doses or alternative medications [22]. To our knowledge, inclusion of this novel haplotype is not available on commercial clinical PGx tests and still requires further clinical validation. However, NGS-based genotype imputation found that more than half of CYP2C19 normal metabolizers in this cohort carry the *CYP2C*-TG haplotype and may benefit from higher dosing or an alternative medication, if validated in subsequent studies. Additional studies are needed to confirm the clinical impact of this novel haplotype on two of the most prescribed antidepressants in this cohort. Additionally, there is emerging evidence for variations in other *CYP450* genes including *CYP2B6* affecting antidepressant concentrations [20, 49]. Incorporating emerging biomarkers into PGx testing guidelines and clinical care in the future will identify more patients with actionable results to guide drug prescribing.

When patients are taking multiple medications, it is important to consider GDDIs which can change the predicted phenotype and medication metabolism. In this cohort, nearly half of patients with medication data were prescribed or being considered for a medication that inhibits CYP2D6, and most (87%) would result in an alternate phenotype due to phenoconversion. Of all patients with medication data, 42% had phenoconversion, which is slightly greater than the 10–30% prevalence seen in other clinical cohorts [50, 51]. This is likely due to this cohort being a psychiatric population where strong CYP2D6-inhibiting medications such as bupropion and fluoxetine are more commonly prescribed. Notably, while patients receiving fluoxetine or paroxetine can experience phenoconversion due to strong CYP2D6 inhibition, the impact of autoinhibition (since these drugs are also metabolized by CYP2D6) is unclear and is likely to be dose-dependent and greater at steady-state concentrations [52]. Nonetheless, these drugs were included in the calculation of proportion with phenoconversion since patients could be on other CYP2D6-metabolized drugs and the overall goal was to determine the proportion experiencing phenoconversion regardless of which drug is causing the inhibition or if autoinhibition is present.

Many laboratory platforms exist for interrogating pharmacogenes, ranging from single-gene assays (e.g., TaqMan) to targeted or WES. While targeted assays are informative for the SNPs of interest, relatively easier to perform with faster turnaround time, and less expensive, the inherent limitations are the inability to accurately detect novel or rare variants not included on the reported panel and inability to disambiguate certain alleles or structural variation within complex genes like *CYP2D6*.

NGS generates data on novel haplotypes which are not measured in most PGx assays. In the largest dataset tested for the *CYP2C*-TG haplotype thus far, we reproduced the overall prevalence and showed that Black patients have a significantly lower frequency of this haplotype compared to Hispanics/Latinos and Whites. Generating data on novel variants across genetic ancestries is important for organizations like the AMP and National Human Genomics Research Institute to expand non-White associations and bring equity to personalized medicine.

There are limitations of this study. First, attempted or considered medication data was only available for a subset of patients, and data may have been missing or affected by reporting bias. This study did not evaluate outcomes related to potential medication changes based on the PGx results and therefore conclusions of clinical utility cannot be made. Only two genes, *CYP2D6* and *CYP2C19*, were evaluated in this study and actionability was limited to CPIC guidelines (2015) [7] and the FDA Table that were available at the time when patients were tested; therefore, more recent CPIC guidelines (2023) [20] or updates to the FDA table could affect the results of this study. Inclusion of additional alleles and GDIs would likely capture additional patients with actionable results to guide medication prescribing. Additionally, the data herein may not be generalizable to PGx tests other than the one used in this study. The alleles evaluated in this study were based on CPIC and AMP guidelines and may report undetected variants as *1, potentially missing unknown actionable phenotypes. Lastly, the combined actionability, including the CYP2C haplotype may be overestimated since the clinical utility of this variant has yet to be confirmed.

## Conclusion

This evaluation of a large real-world psychiatric patient population receiving clinical PGx testing identified a high proportion of patients with potential actionability for medication prescribing decisions. While this analysis focused on two genes (*CYP2D6* and *CYP2C19*), other emerging genes may also play a role and further increase the potential actionability of PGx testing. This study underscores the potential value of using NGS to detect CYP2D6 allele-specific CNV allele assignment in *CYP2D6* genotype/phenotype calling and the novel CYP2C haplotype. When accounting for these variations, as well as phenoconversion, most patients displayed some level of actionability. This information is critical for clinicians, laboratories, and researchers to consider when applying PGx in psychiatric care.

## Supplementary information

Supplement

Supplementary Figure S1. Ancestry admixtures by imputed race/ethnicity

## Data Availability

Deidentified data used in the research was collected in a real-world healthcare setting and is subject to controlled access for privacy and proprietary reasons. When possible, derived data supporting the findings of this study have been made available within the paper and its Supplementary figures/tables.
