# Supplementary material for: Pharmacogenomic insights in psychiatric care: uncovering novel actionability, allele-specific CYP2D6 copy number variation, and phenoconversion in 15,000 patients
Source: Mol Psychiatry. 2024 May 23;29(11):3495–502. doi: 10.1038/s41380-024-02588-4 (PMC11541190; doi:10.1038/s41380-024-02588-4)
Supplement: Supplementary file 1 — Supplement [file 41380_2024_2588_MOESM1_ESM.docx]

# Online-Only Supplement

**Supplementary Table S1.** PGx Actionability Guidance from the FDA

**Supplementary Table S2.** CYP2D6 Phenoconversion based on Flockhart Table

**Supplementary Table S3**. Clinical actionability for medications attempted

**Supplementary Table S4**. Clinical actionability for medications considered at the time of PGx testing

**Supplementary Table S5.** Clinical actionability for medication classes attempted at the time of PGx testing

**Supplementary Table S6.** Clinical actionability for medication classes considered at the time of PGx testing

**Supplementary Figure S1.** Ancestry admixtures by imputed race/ethnicity

**Supplementary Table S7**. Frequency of CNVs by imputed race/ethnicity

**Supplementary Table S8.** Phenoconversion of patients who have taken or are considering CYP2D6 inhibitor medications with different strengths

**Supplementary Table S9**. Frequency of CYP2C TG haplotype actionability by imputed race/ethnicity

**Supplementary Table S10**. Pairwise proportion tests for CYP2C-TG carrier frequency differences between imputed race/ethnicities

**Supplementary Table S11**. Pairwise proportion tests for different rates of CYP2C-TG actionability change between imputed race/ethnicities

**Supplementary Methods**

**Supplementary Results**

**Supplementary Table S1. PGx Actionability Guidance from the FDA and CPIC** [[1–4]](https://paperpile.com/c/yuUYhO/gPSx+LLOW+6Ygx+Uxwq)

| **Gene** | **Phenotype** | **Medication** | **Source** |
| --- | --- | --- | --- |
| CYP2C19 | Ultrarapid | amitriptyline | CPIC |
| CYP2C19 | Ultrarapid | citalopram | CPIC |
| CYP2C19 | Ultrarapid | escitalopram | CPIC |
| CYP2C19 | Ultrarapid | clomipramine | FDA |
| CYP2C19 | Ultrarapid | doxepin | CPIC |
| CYP2C19 | Ultrarapid | imipramine | CPIC |
| CYP2C19 | Rapid | amitriptyline | CPIC |
| CYP2C19 | Rapid | citalopram | CPIC |
| CYP2C19 | Rapid | escitalopram | CPIC |
| CYP2C19 | Rapid | clomipramine | CPIC |
| CYP2C19 | Rapid | doxepin | CPIC |
| CYP2C19 | Rapid | imipramine | CPIC |
| CYP2C19 | Poor | amitriptyline | CPIC |
| CYP2C19 | Poor | citalopram | CPIC, FDA |
| CYP2C19 | Poor | escitalopram | CPIC |
| CYP2C19 | Poor | clomipramine | CPIC |
| CYP2C19 | Poor | doxepin | CPIC |
| CYP2C19 | Poor | imipramine | CPIC |
| CYP2C19 | Poor | sertraline | CPIC |
| CYP2C19 | Poor | clobazam | FDA |
| CYP2C19 | Poor | brivaracetam | FDA |
| CYP2D6 | Ultrarapid | amitriptyline | CPIC |
| CYP2D6 | Ultrarapid | imipramine | CPIC |
| CYP2D6 | Ultrarapid | clomipramine | CPIC |
| CYP2D6 | Ultrarapid | desipramine | CPIC |
| CYP2D6 | Ultrarapid | doxepin | CPIC |
| CYP2D6 | Ultrarapid | nortriptyline | CPIC |
| CYP2D6 | Ultrarapid | paroxetine | CPIC |
| CYP2D6 | Intermediate | amitriptyline | CPIC |
| CYP2D6 | Intermediate | imipramine | CPIC |
| CYP2D6 | Intermediate | clomipramine | CPIC |
| CYP2D6 | Intermediate | desipramine | CPIC |
| CYP2D6 | Intermediate | doxepin | CPIC |
| CYP2D6 | Intermediate | nortriptyline | CPIC |
| CYP2D6 | Poor | amitriptyline | CPIC |
| CYP2D6 | Poor | imipramine | CPIC |
| CYP2D6 | Poor | clomipramine | CPIC |
| CYP2D6 | Poor | desipramine | CPIC |
| CYP2D6 | Poor | doxepin | CPIC |
| CYP2D6 | Poor | nortriptyline | CPIC |
| CYP2D6 | Poor | fluvoxamine | CPIC |
| CYP2D6 | Poor | paroxetine | CPIC |
| CYP2D6 | Poor | aripiprazole | FDA |
| CYP2D6 | Poor | brexpiprazole | FDA |
| CYP2D6 | Poor | vortioxetine | FDA |
| CYP2D6 | Poor | thioridazine | FDA |
| CYP2D6 | Poor | amphetamine | FDA |
| CYP2D6 | Poor | atomoxetine | FDA |
| CYP2D6 | Poor | clozapine | FDA |
| CYP2D6 | Poor | deutetrabenazine | FDA |
| CYP2D6 | Poor | iloperidone | FDA |
| CYP2D6 | Poor | pitolisant | FDA |
| CYP2D6 | Poor | valbenazine | FDA |
| CYP2D6 | Poor | venlafaxine | FDA |

**Supplementary Table S2. CYP2D6 Inhibitors based on Flockhart Table** [[5]](https://paperpile.com/c/yuUYhO/O1Za)

| **Medication** | **CYP2D6 Inhibitor Strength** |
| --- | --- |
| abiraterone | moderate |
| bupropion | strong |
| cinacalcet | moderate |
| duloxetine | moderate |
| fluoxetine | strong |
| lorcaserin | moderate |
| mirabegron | moderate |
| paroxetine | strong |
| quinidine | strong |
| rolapitant | moderate |
| terbinafine | strong |

**Supplementary Table S3. Clinical actionability for medications attempted.**

| **Medication** | **No. of patients** | **No. of patients actionable** | **Fraction actionable (%)** |
| --- | --- | --- | --- |
| escitalopram | 556 | 182 | 33 |
| citalopram | 188 | 57 | 30 |
| amitriptyline | 62 | 43 | 69 |
| doxepin | 34 | 26 | 76 |
| aripiprazole | 377 | 23 | 6 |
| venlafaxine | 283 | 17 | 6 |
| sertraline | 670 | 16 | 2 |
| amphetamine | 326 | 15 | 5 |
| nortriptyline | 27 | 15 | 56 |
| clomipramine | 16 | 14 | 88 |
| paroxetine | 132 | 10 | 8 |
| vortioxetine | 128 | 8 | 6 |
| atomoxetine | 119 | 7 | 6 |
| imipramine | 11 | 7 | 64 |
| brexpiprazole | 57 | 4 | 7 |
| desipramine | 4 | 3 | 75 |
| fluvoxamine | 49 | 1 | 2 |
| valbenazine | 4 | 1 | 25 |
| clozapine | 7 | 0 | 0 |
| thioridazine | 5 | 0 | 0 |
| deutetrabenazine | 2 | 0 | 0 |
| clobazam | 1 | 0 | 0 |
| iloperidone | 1 | 0 | 0 |

**Supplementary Table S4. Clinical actionability for medications considered at the time of PGx testing.**

| **Medication** | **No. of patients** | **No. of patients actionable** | **Fraction actionable (%)** |
| --- | --- | --- | --- |
| escitalopram | 425 | 134 | 32 |
| citalopram | 158 | 48 | 30 |
| amitriptyline | 41 | 27 | 66 |
| amphetamine | 231 | 19 | 8 |
| doxepin | 37 | 18 | 49 |
| sertraline | 552 | 16 | 3 |
| aripiprazole | 319 | 13 | 4 |
| venlafaxine | 274 | 12 | 4 |
| atomoxetine | 127 | 12 | 9 |
| paroxetine | 126 | 11 | 9 |
| vortioxetine | 219 | 10 | 5 |
| nortriptyline | 30 | 10 | 33 |
| clomipramine | 21 | 10 | 48 |
| brexpiprazole | 112 | 4 | 4 |
| fluvoxamine | 73 | 4 | 5 |
| imipramine | 3 | 2 | 67 |
| desipramine | 2 | 2 | 100 |
| clozapine | 21 | 1 | 5 |
| iloperidone | 2 | 0 | 0 |
| deutetrabenazine | 1 | 0 | 0 |
| valbenazine | 1 | 0 | 0 |

**Supplementary Table S5. Clinical actionability for medication classes attempted at the time of PGx testing.**

| **Therapy Class** | **No. of attempted medications** | **No. of patients** | **Fraction of medications actionable (%)** |
| --- | --- | --- | --- |
| SSRI | 1595 | 1190 | 17 |
| Second Generation | 442 | 422 | 6 |
| Stimulant | 326 | 326 | 5 |
| SNRI | 283 | 283 | 6 |
| TCA | 154 | 132 | 70 |
| Other Antidepressants | 128 | 128 | 6 |
| Non-stimulant | 119 | 119 | 6 |
| Other | 6 | 6 | 17 |
| First Generation | 5 | 5 | 0 |
| Benzodiazepine | 1 | 1 | 0 |

**Supplementary Table S6. Clinical actionability for medication classes considered at the time of PGx testing.**

| **Therapy Class** | **No. of considered medications** | **No. of patients** | **Fraction actionable (%)** |
| --- | --- | --- | --- |
| SSRI | 1334 | 990 | 16 |
| Antipsychotic | 454 | 423 | 4 |
| SNRI | 274 | 274 | 4 |
| Stimulant | 231 | 231 | 8 |
| Other Antidepressants | 219 | 219 | 5 |
| TCA | 134 | 123 | 52 |
| Non-stimulant | 127 | 127 | 9 |
| Other | 2 | 1 | 0 |

SSRI: selective serotonin reuptake inhibitor, SNRI: serotonin norepinephrine reuptake inhibitor, TCA: tricyclic antidepressants

**Supplementary Figure S1. Ancestry admixtures by imputed race/ethnicity in 15,000 patients**

**Supplementary Figure S1**. Each column represents a patient’s genetic ancestry proportions. Based on genetic ancestry proportions, race/ethnicity imputation predicts how the patient would be expected to self-identify, indicated by the x-axis labels. Patients with no majority ancestry are grouped into an “Admixed” race/ethnicity group.

**Supplementary Table S7. Frequency of CNVs by imputed race/ethnicity.**

| **Race/Ethnicity** | **No. of patients** | **No. with CNVs** | **Fraction w/ CNVs** | **No. with Allele-Assignment-Dependent** | **Fraction Allele-Assignment-Dependent (%)** |
| --- | --- | --- | --- | --- | --- |
| White | 12,250 | 741 | 0.06 | 259 | 2 |
| Hispanic/Latino | 1,077 | 78 | 0.07 | 18 | 2 |
| Black | 964 | 122 | 0.13 | 44 | 5 |
| Admixed | 371 | 62 | 0.17 | 11 | 3 |
| Asian | 338 | 101 | 0.30 | 6 | 2 |

**Supplementary Table S8. Phenoconversion of patients who have taken or are considering *CYP2D6* inhibitor medications with varying strengths.**

| **Genetic metabolism** | **CYP2D6 inhibition** | **No. of patients** | **effective metabolism** | **Is metabolism changed** |
| --- | --- | --- | --- | --- |
| Normal | strong | 888 | Poor | yes |
| Intermediate | strong | 648 | Poor | yes |
| Normal | moderate | 126 | Intermediate | yes |
| Intermediate | moderate | 110 | Intermediate | no |
| Poor | strong | 120 | Poor | no |
| Ultrarapid | strong | 67 | Poor | yes |
| Poor | moderate | 18 | Poor | no |
| Ultrarapid | moderate | 5 | Normal | yes |

**Supplementary Table S9. Frequency of *CYP2C* TG haplotype actionability by imputed race/ethnicity.**

| **Race/Ethnicity** | **No. of Patients** | **Percent with CYP2C-TG (%)** | **Percent with CYP2C19 *1/*1 (%)** | **Percent with altered Phenotypes (%)** |
| --- | --- | --- | --- | --- |
| Hispanic/Latino | 253 | 46 | 49 | 30 |
| Admixed | 85 | 44 | 38 | 21 |
| White | 3,484 | 37 | 39 | 21 |
| Asian | 80 | 35 | 30 | 19 |
| Black | 212 | 15 | 37 | 9 |

**Supplementary Table S10. Pairwise proportion tests for *CYP2C*-TG carrier frequency differences between imputed race/ethnicities.**

| **Race/Ethnicity 1** | **Race/Ethnicity 2** | **Adjusted p-value** |
| --- | --- | --- |
| Hispanic/Latino | Black | 3e-11 |
| White | Black | 3e-9 |
| Black | Admixed | 3e-6 |
| Black | Asian | 2e-3 |
| Hispanic/Latino | White | 3e-02 |

**Supplementary Table S11.** **Pairwise proportion tests for different rates of CYP2C-TG actionability change between imputed race/ethnicities.**

| **Race/Ethnicity 1** | **Race/Ethnicity 2** | **adjusted p-value** |
| --- | --- | --- |
| Hispanic/Latino | Black | 9e-17 |
| White | Black | 1e-3 |
| White | Hispanic/Latino | 4e-3 |
| Black | Admixed | 8e-2 |
| Hispanic/Latino | White | 3e-02 |

##

##

##

##

##

##

##

## **Supplementary Methods**

### **Sequencing Equipment, Bioinformatic Processing, and Validation**

Tempus nP includes a combination of targeted hybridization pull-down capture and NGS sequencing with Illumina 2x150bp paired end libraries in the NovaSeq 6000 (Illumina, San Diego California), and MassARRAY^®^ (Agena Biosciences, San Diego California) based quantitative genotyping. The targeted sequencing capture assay includes a WES backbone with custom probes added to increase depth of coverage around the PGx genes of interest. Reads were aligned to the human reference genome (hg19) with the bioinformatics software BWA and variant calling was performed with GATK HaplotypeCaller. An average of 116,661 variants in exons or regions targeted by the assay are called; and the mean target exon read depth was 62x (interquartile range 52-72x; estimated with 100 random files).

Variant call data was processed into preliminary star allele and metabolizer phenotypes by in-house software. The PGx assay has been analytically validated for *CYP2C19* alleles *1, *2, *3, *4A, *4B, *5 *6, *7 ,*8, *9, *10, *17, and *35, and for *CYP2D6* alleles *1, *1xN, *2, *2xN, *3, *3xN, *4, *4xN, *5, *6, *6xN, *7, *8, *9, *9xN, *10, *10xN, *11, *12, *14, *15, *17,*17xN, *29, *29xN, *35, *35xN, *40, *41, and *41xN.

###

###

###

###

###

###

###

###

###

###

###

### **Haplotype Imputation**

The imputation software, GLIMPSE,[[6]](https://paperpile.com/c/yuUYhO/1EsrO) leverages low-coverage NGS data with a genetic map and a haplotype reference panel in order to impute and phase genotypes for a patient sample at every polymorphic site in the reference panel. In this study, GLIMPSE utilized for imputation: 1) low-coverage NGS sequencing data from nP off-target reads for each patient, 2) a genetic recombination rate map from HapMapII ([data](http://ftp.1000genomes.ebi.ac.uk/vol1/ftp/technical/working/20110106_recombination_hotspots/HapmapII_GRCh37_RecombinationHotspots.tar.gz)),[[7]](https://paperpile.com/c/yuUYhO/HiHp2) and 3) a panel of reference haplotypes from the 1000 Genomes Project (TGP) phase 3[[8]](https://paperpile.com/c/yuUYhO/xW5LN) ([data](http://ftp.1000genomes.ebi.ac.uk/vol1/ftp/release/20130502/)). The NIST sample, HG001, was removed during subsequent performance benchmarking of imputation results (described below), but was kept for the genotype imputation of patient samples. The GLIMPSE imputation algorithm cannot properly utilize loci with >2 alleles (i.e., non-bi-allelic), therefore these sites were excluded from imputation. Further, from the patient off-target reads NGS data, GLIMPSE utilizes genotype likelihoods as the primary inputs. These likelihoods were generated using bcftools at each non-bi-allelic SNP position in the TGP reference panel.[[9, 10]](https://paperpile.com/c/yuUYhO/G0au+LyOQ) However, since bcftools generates poor genotype likelihoods for insertions/deletions, these were imputed using uniform genotype likelihoods. All TGP reference panel variants (minus non-bi-allelic sites) in the genomic region of interest, chr10:96375267-98547144 (build GRCh37), were imputed. An additional 200 kb of buffer bases were also added to the flanks of the region of interest in order to avoid low-quality edge effects.

To characterize the parameters that contribute to imputation performance, we also measured the number of sites in the TGP reference panel that were directly sequenced by nP NGS off-target reads and the sequencing coverage at those sites, which is known as the “effective coverage” [[11]](https://paperpile.com/c/yuUYhO/H2Yz).

###

###

###

###

###

### **Imputation commands**

# compute SNP GT likelihoods of off-target reads with bcftools

# (example here for region of interest 10:96375267-98547144)

bcftools mpileup -f hs37d5.fa.gz -I -E -a 'FORMAT/DP' -T \

chr10_TGP_SNPs.vcf.gz -r 10 input_sample.bam -Ou | bcftools call -Aim -C \ alleles -T chr10_TGP_SNPs.tsv.gz -Oz -o chr10_TGP_SNPs_GT_likelihoods.vcf.gz

# run genotype imputation with GLIMPSE across region of interest

# (10:96375267-98547144)

# NOTE: input chr10_TGP_GT_likelihoods.vcf.gz includes indels with uniform GT # likelihoods

GLIMPSE_phase --input chr10_TGP_GT_likelihoods.vcf.gz --reference \

chr10_TGP.bcf --map genetic_map_GRCh37_chr10.txt.gz --main 15 --input-region \

"10:96175228-98747177" --output-region "10:96375267-98547144" --output \ chr10_96375267_98547144_imputed_sample.bcf

# phase imputed genotypes with GLIMPSE and convert output to vcf.gz

GLIMPSE_sample --input chr10_96375267_98547144_imputed_sample.bcf --solve \ --output chr10_96375267_98547144_imputed_sample.phased.bcf

bcftools index -f chr10_96375267_98547144_imputed_sample.phased.bcf

bcftools view chr10_96375267_98547144_imputed_sample.phased.bcf -Oz -o \ chr10_96375267_98547144_imputed_sample.phased.vcf.gz

bcftools index -f -t chr10_96375267_98547144_imputed_sample.phased.vcf.gz

###

###

###

###

###

###

###

###

###

### **Haplotype Imputation Benchmarking**

We performed haplotype imputation benchmarking using the HG001 reference human cell line (also known as Coriell Institute cell line NA12878) characterized by the NIST Genome-in-a-Bottle (GiaB) consortium. DNA from the HG001 lymphoblastoid cell line obtained from the Coriell Institute (Camden, NJ) is sequenced on every flow cell for the nP assay as a quality control standard. For HG001, there is also a high-quality variant truth data set released by GiaB that can be used for performance benchmarking (version 4.2.1; data: <https://ftp.ncbi.nlm.nih.gov/ReferenceSamples/giab/release/NA12878_HG001/NISTv4.2.1/GRCh37/>) [[12]](https://paperpile.com/c/yuUYhO/XtHf). We ran imputation on 8 nP HG001 sample replicates (1 reference patient specimen, 8 sequencing reactions/vials, 4 flow-cells) and checked prediction accuracy by comparing imputed genotypes to the GiaB high-quality benchmark using best practices put forward by the Global Alliance for Genomics and Health Benchmarking Team [[13]](https://paperpile.com/c/yuUYhO/2AMR).

We first evaluated imputation performance on the 2 loci that contribute to the novel *CYP2C* haplotype: rs2860840C>T and rs11188059G>A. We report the accuracy at each locus as the number of samples predicted to have the same genotypes as found in the GiaB high-quality benchmark.

We then measured imputation performance on millions of common variants across the whole genome. Specifically, common variants on chr1-22 (i.e., all bi-allelic variants found in the TGP reference panel for chr1-22; N=81,646,132 variant sites) were imputed for HG001 and the results were compared to the GiaB high-quality benchmark. 3,891,440 sites in the HG001 GiaB high-quality benchmark are characterized as polymorphic on chr1-22. We then measured the genome-wide imputation performance by calculating precision and sensitivity for each imputed sample replicate and reported the mean and extreme performance observations across the samples.

##

##

##

##

## **Supplementary Results**

Imputation performance was evaluated using the HG001 high-quality benchmark. The HG001 high-quality benchmark has reference genotypes of 0/0 and 0/1 at rs2860840 and rs11188059, respectively, and the 8 imputed nP HG001 samples were all predicted to have 0/0 and 0/1 at rs2860840 and rs11188059 (100% accuracy, N=8 sequencing replicates). Whole-genome (chr1-22) imputation was used to predict genotypes at ~82 million sites in HG001 sequencing replicates, of which ~3.9 million are characterized as polymorphic in the high-quality benchmark. For the whole-genome results across the 8 sequencing replicates, the mean positive predictive value (PPV) for imputed genotypes was observed to be 85% (the worst and best replicates were 83% and 86%) and mean sensitivity was observed to be 75% (the worst and best replicates were 73% and 76%).

##

##

##

## **References**

1. [Center for Devices, Radiological Health. Table of Pharmacogenetic Associations. US Food and Drug Administration.](http://paperpile.com/b/yuUYhO/gPSx) <https://www.fda.gov/medical-devices/precision-medicine/table-pharmacogenetic-associations.> [Accessed 20 March 2023.](http://paperpile.com/b/yuUYhO/gPSx)

2. [Hicks JK, Sangkuhl K, Swen JJ, Ellingrod VL, Müller DJ, Shimoda K, et al. Clinical pharmacogenetics implementation consortium guideline (CPIC) for CYP2D6 and CYP2C19 genotypes and dosing of tricyclic antidepressants: 2016 update. Clin Pharmacol Ther. 2017;102:37–44.](http://paperpile.com/b/yuUYhO/LLOW)

3. [Hicks JK, Bishop JR, Sangkuhl K, Müller DJ, Ji Y, Leckband SG, et al. Clinical Pharmacogenetics Implementation Consortium (CPIC) Guideline for CYP2D6 and CYP2C19 Genotypes and Dosing of Selective Serotonin Reuptake Inhibitors. Clin Pharmacol Ther. 2015;98:127–134.](http://paperpile.com/b/yuUYhO/6Ygx)

4. [Brown JT, Bishop JR, Sangkuhl K, Nurmi EL, Mueller DJ, Dinh JC, et al. Clinical Pharmacogenetics Implementation Consortium Guideline for Cytochrome P450 (CYP)2D6 Genotype and Atomoxetine Therapy. Clin Pharmacol Ther. 2019;106:94–102.](http://paperpile.com/b/yuUYhO/Uxwq)

5. [Indiana University. Drug Interactions Flockhart Table. 2024.](http://paperpile.com/b/yuUYhO/O1Za) <https://drug-interactions.medicine.iu.edu/MainTable.aspx.> [Accessed 16 February 2024.](http://paperpile.com/b/yuUYhO/O1Za)

6. [Rubinacci S, Ribeiro DM, Hofmeister RJ, Delaneau O. Efficient phasing and imputation of low-coverage sequencing data using large reference panels. Nat Genet. 2021;53:120–126.](http://paperpile.com/b/yuUYhO/1EsrO)

7. [International HapMap Consortium, Frazer KA, Ballinger DG, Cox DR, Hinds DA, Stuve LL, et al. A second generation human haplotype map of over 3.1 million SNPs. Nature. 2007;449:851–861.](http://paperpile.com/b/yuUYhO/HiHp2)

8. [1000 Genomes Project Consortium, Auton A, Brooks LD, Durbin RM, Garrison EP, Kang HM, et al. A global reference for human genetic variation. Nature. 2015;526:68–74.](http://paperpile.com/b/yuUYhO/xW5LN)

9. [Li H. A statistical framework for SNP calling, mutation discovery, association mapping and population genetical parameter estimation from sequencing data. Bioinformatics. 2011;27:2987–2993.](http://paperpile.com/b/yuUYhO/G0au)

10. [Danecek P, Bonfield JK, Liddle J, Marshall J, Ohan V, Pollard MO, et al. Twelve years of SAMtools and BCFtools. Gigascience. 2021;10.](http://paperpile.com/b/yuUYhO/LyOQ)

11. [Li JH, Mazur CA, Berisa T, Pickrell JK. Low-pass sequencing increases the power of GWAS and decreases measurement error of polygenic risk scores compared to genotyping arrays. Genome Res. 2021;31:529–537.](http://paperpile.com/b/yuUYhO/H2Yz)

12. [Zook JM, McDaniel J, Olson ND, Wagner J, Parikh H, Heaton H, et al. An open resource for accurately benchmarking small variant and reference calls. Nat Biotechnol. 2019;37:561–566.](http://paperpile.com/b/yuUYhO/XtHf)

13. [Krusche P, Trigg L, Boutros PC, Mason CE, De La Vega FM, Moore BL, et al. Best practices for benchmarking germline small-variant calls in human genomes. Nat Biotechnol. 2019;37:555–560.](http://paperpile.com/b/yuUYhO/2AMR)
