# Supplementary figures and images for: Pharmacogenomic insights in psychiatric care: uncovering novel actionability, allele-specific CYP2D6 copy number variation, and phenoconversion in 15,000 patients
Source: Mol Psychiatry. 2024 May 23;29(11):3495–502. doi: 10.1038/s41380-024-02588-4 (PMC11541190; doi:10.1038/s41380-024-02588-4)

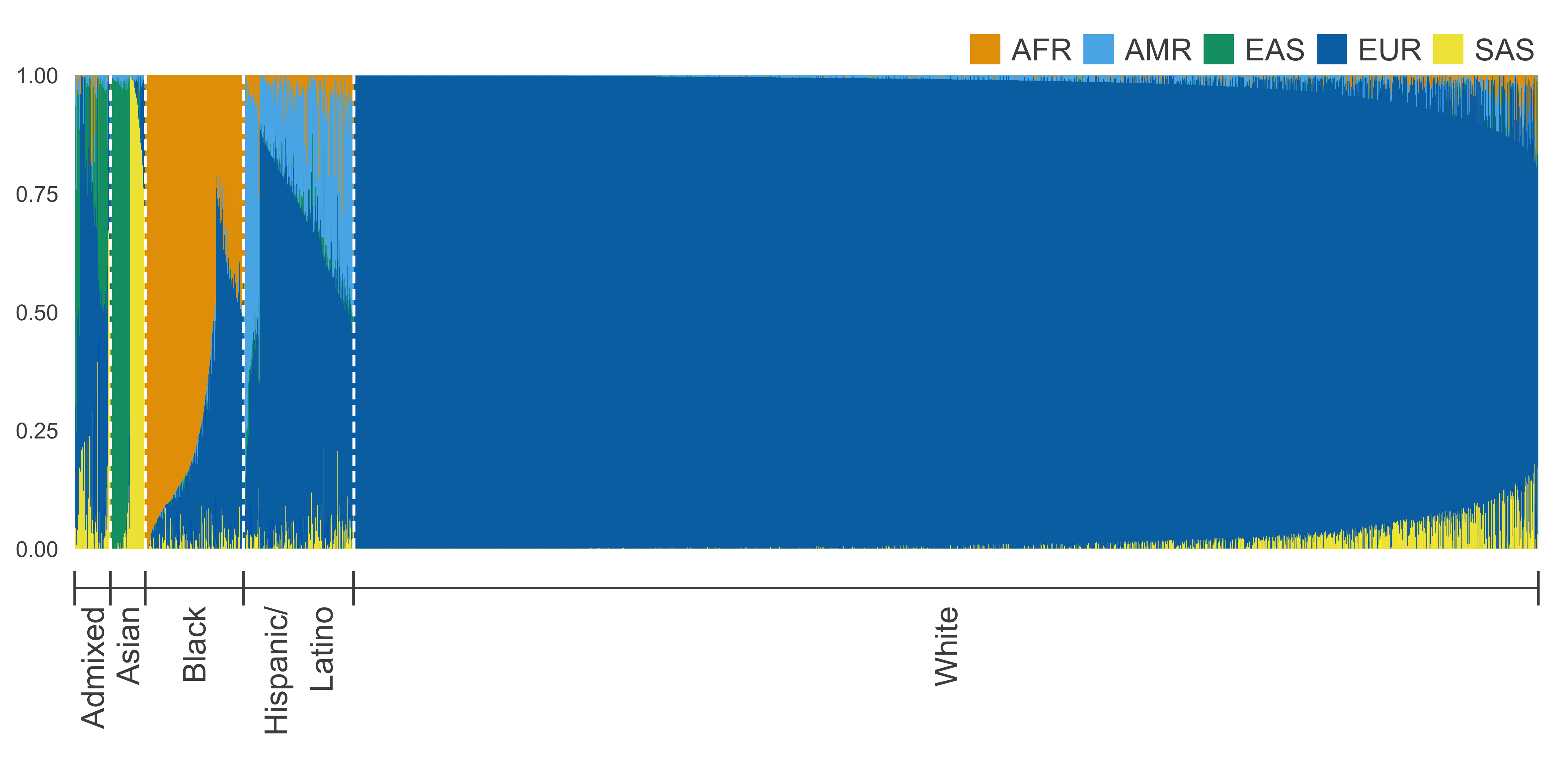

Supplement: Supplementary file 2 — Supplementary Figure S1. Ancestry admixtures by imputed race/ethnicity [file 41380_2024_2588_MOESM2_ESM.tif]
